# Characteristics analysis of segmental and regional lumbar spontaneous compensation post thoracic fusion in Lenke 1 and 2 adolescent idiopathic scoliosis

**DOI:** 10.1186/s12891-021-04821-5

**Published:** 2021-11-10

**Authors:** Kai Chen, Xiao Zhai, Tianjunke Zhou, Yu Deng, Beichen Zhang, Shaofeng Chen, Changwei Yang, Ming Li

**Affiliations:** 1 Department of Orthopedics, Shanghai Changhai Hospital, No. 168, Changhai Road, Shanghai, 200433 China; 2 Basic medicine college, Navy Medical University, Shanghai, 200433 China; 3 Faculty of Anesthesiology, Shanghai Changhai Hopital, Shanghai, 200433 China; 4 Department of Rehabilitation, The First Rehabilitation Hospital of Shanghai, Shanghai, 200082 China

**Keywords:** Thoracic fusion, AIS, Spontaneous compensation, Characteristics, Unfused lumbar segments

## Abstract

**Objective:**

To explore the characteristics of compensation of unfused lumbar region post thoracic fusion in Lenke 1 and 2 adolescent idiopathic scoliosis.

**Background:**

Preserving lumbar mobility in the compensation is significant in controlling pain and maintaining its functions. The spontaneous correction of the distal unfused lumbar curve after STF has been widely reported, but previous study has not concentrated on the characteristics of compensation of unfused lumbar region post thoracic fusion.

**Method:**

A total of 51 Lenke 1 and2 AIS patients were included, whose lowest instrumented vertebrae was L1 from January 2013 to December 2019. For further analysis, demographic data and coronal radiographic films were collected before surgery, at immediate erect postoperatively and final follow-up. The wedge angles of each unfused distal lumbar segments were measured, and the variations in each disc segment were calculated at the immediate postoperative review and final follow-up. Meanwhile, the unfused lumbar curve was divided into upper and lower parts, and we calculated their curve angles and compensations.

**Results:**

The current study enrolled 41 females (80.4%) and 10 males (19.6%). Thirty-six patients were Lenke type 1, while 15 patients were Lenke type 2. The average main thoracic Cobb angle and thoracolumbar/lumbar Cobb angle were 44.1 ± 7.7°and 24.1 ± 9.3°, preoperatively. At the final follow-up, the disc wedge angle variation of L1/2, L2/3, L3/4, L4/5 and L5/S1 was 3.84 ± 5.96°, 3.09 ± 4.54°, 2.30 ± 4.53°, − 0.12 ± 3.89° and − 1.36 ± 2.80°, respectively. The compensation of upper and lower coronal lumbar curves at final follow-up were 9.22 ± 10.39° and − 1.49 ± 5.14°, respectively.

**Conclusion:**

When choosing L1 as the lowest instrumented vertebrae, the distal unfused lumbar segments’ compensation showed a decreasing trend from the proximal end to the distal end. The adjacent L1/2 and L2/3 discs significantly contributed to this compensation.

## Background

Lenke 1 and 2 adolescent idiopathic scoliosis (AIS) accounts for most AIS, but the optimal therapeutic approach for these patients remains controversial [1]. As posterior selective thoracic fusion (STF) has become the standard surgical treatment of choice [2], numerous studies have shown that proper STF can accomplish the underlying aim of preventing scoliosis progression while maintaining global balance [3–8]. In addition, the spontaneous lumbar curve correction (SLCC) can be achieved by correcting the main thoracic curve. In spinal fusion, it is believed that preserving lumbar mobility is advantageous in controlling pain and maintaining its functions. However, when planning the surgical treatments, more attention should be paid to the patients’ unfused lumbar curve compensation ability due to its importance to the coronal balance. Otherwise, the compensation characteristics of the spontaneous distal lumbar curve remain unclear even though it has been mentioned in some articles.

Bachmann et al. [9] validated that STF mainly produced changes in the upper half of the lumbar curve, leaving the lower half and the lumbosacral takeoff angle with little change. Mason et al. [10] proposed that most lumbar coronal corrections could occur in the proximal region above the lumbar apex post STF. They explained that the proximal lumbar coronal curve could be more significantly corrected than the distal lumbar area because the proximal lumbar curve would become more lordotic in the sagittal plane immediately after surgery [11]. Meanwhile, with a more distal lowest instrumented vertebrae (LIV), there is increased disc pressure and segmental motion at the adjacent level, followed by an overall reduction in lumbar activity and an increased risk of disc degeneration. Meric et al. [12] conducted a retrospective study of AIS patients who received STF treatment, with at least 10 years follow-up demonstrated a moderate rise in disc degeneration in the unfused segments. Facet joint degeneration was significant at the upper two levels adjacent to the lowest instrumented vertebra.

Although the spontaneous correction of the distal unfused lumbar curve after STF has been widely reported [1, 13–20]; however, the impact of the spontaneous realignment of unfused segments on disc compensation remains to be quantified. To obtain an optimal balance outcome and prevent radiographical complications, such as the adding-on phenomenon, research has been conducted regarding the optimal LIV selection [8, 21], prediction of SLCC [14, 15, 17] and related long-term outcome [6, 22]. However, all these studies regarded the unfused distal segments as an ensemble. Till, no further research has been reported on the impact of segmental or regional disc variation. Our study focused on the distribution of distal unfused lumbar disc variation and explored the characteristics of compensation of unfused lumbar region post thoracic fusion in Lenke 1 and 2 adolescent idiopathic scoliosis.

## Methods

### Patients populations

**Fig. 1:**
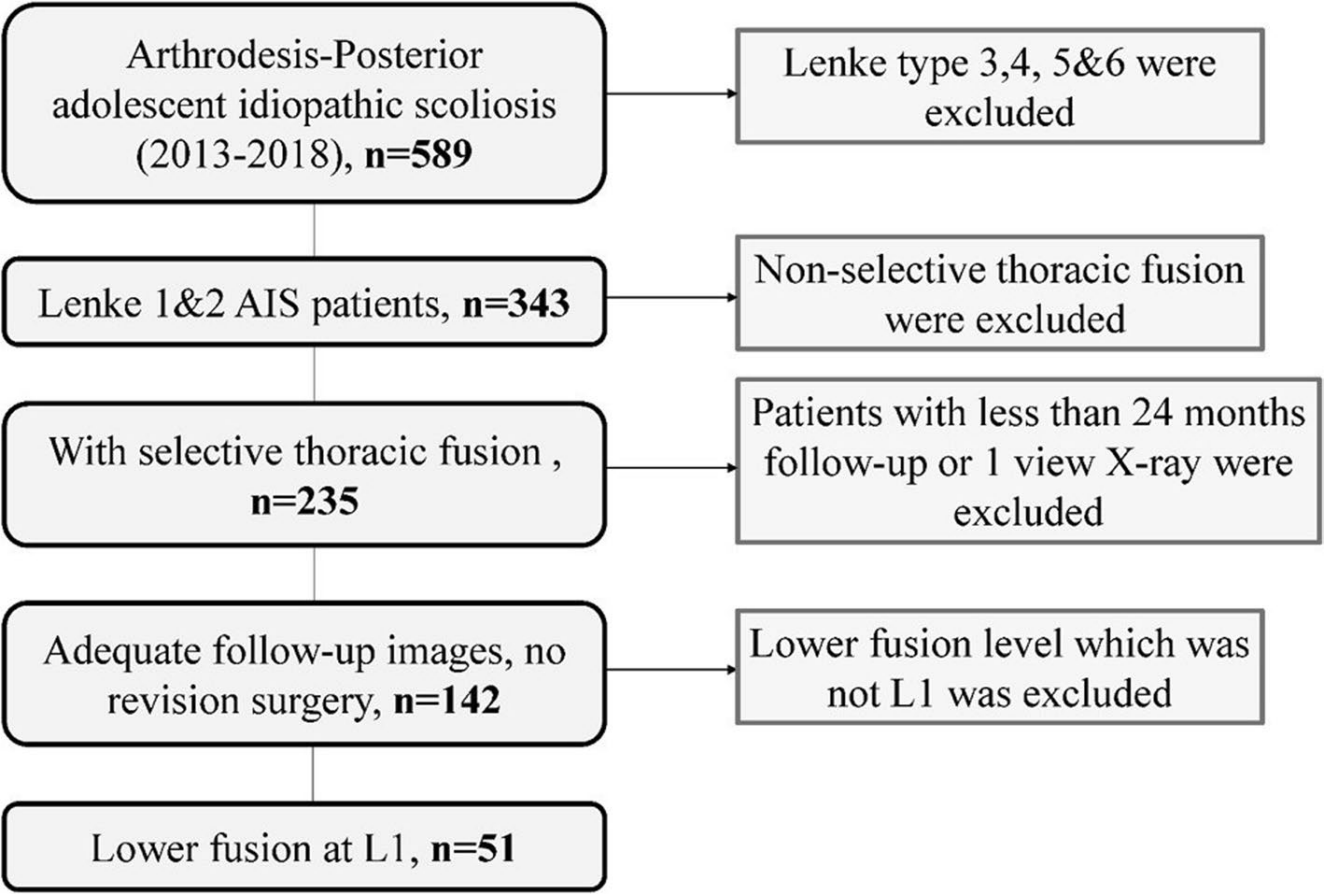
Flow chart showing the process of selection

A total of 51 patients were enrolled in this study between January 2013 and December 2019 met the inclusion and exclusion criteria as shown in Fig. 1. The inclusion criteria were: 1). 10 ≤ Age ≤ 18 years old; 2). According to Lenke classification, patients were diagnosed with Lenke 1 and 2 AIS and received a one-stage posterior correction surgery with pedicle screw; 3). LIV was L1 vertebrae; 4). The total follow-up time exceeded 24 months. The exclusion criteria were: 1). Other types of AIS or spine deformity; 2). LIV was above or below L1 vertebrae. In addition, patients without adequate radiological materials were also excluded. This current study was approved by the institutional review board of our hospital, and the patients in our study provided written informed consent for the study.

### Data collection

The demographic data, including age, gender, height, weight, BMI and Lenke type were recorded. Surgery-related information was recorded, such as UIV (upper instrumented vertebrae), fusion segments, and pedicle screws. All patients provided full spine standing posterior-anterior X-ray before surgery, at the immediate postoperative follow-up and final follow-up. The Risser sign was calculated according to the preoperative pelvic X-ray. Other radiographic parameters were measured using Surgimap software, such as proximal thoracic Cobb angles, main thoracic Cobb angles, thoracolumbar/lumbar Cobb angles, translation of thoracic apex (TAVT, the distance between the apex vertebra of the main thoracic curve and the cervical 7 vertebrae plumb line (C7PL)), translation of thoracolumbar/lumbar apex (LAVT, the distance between the apex vertebra of the thoracolumbar/lumbar curve and the center sacral vertical line (CSVL)) and coronal balance (the horizontal distance between the CSVL drawn from C7PL). The disc wedge angle was measured as the angle between the lines along the inferior endplate of the upper and the superior endplate of the lower vertebra in a segment, L1/2, L2/3, L3/4, L4/5 and L5/S1 disc were measured, respectively. Each segment’s variation of disc wedge angle was calculated at immediate postoperative follow-up and final follow-up reviews. As for the analysis of integral distal lumbar compensation, upper coronal lumbar curve (the Cobb angle between L1 and L4) and lower coronal lumbar curve (the Cobb angle between L4 and S1) were measured, and their compensation ability was also calculated at each follow-up. Radiographic parameters were measured by two experienced attending doctors of spine deformity (Dr X. Z. and Dr. K. C.), and the average value was adopted for further analysis.

### Statistical analysis

Statistical analysis was performed using SPSS 19.0 statistics software (SPSS Inc., Chicago, IL). Descriptive statistics were presented in the form of mean ± standard deviation (SD). ANOVA analysis was used to assess the quantitative data among different periods, and the SNK method was used for pairwise comparison. Pair t-test analysis was utilized to assess the compensation ability of the upper and lower coronal lumbar curve. Correlation analysis was also adopted to clarify the composition and compensation ability of each segment in the whole unfused lumbar region. *P* < 0.05 was considered statistical significance.

## Results

A total of 51 Lenke 1 and 2 patients were enrolled in our study, including 41 females (80.4%) and 10 males (19.6%). Thirty-six patients were Lenke type 1, while 15 patients were Lenke type 2. The mean age at the time of surgery was 14.12 ± 2.05 years. The average preoperative height was 159.33 ± 6.85 cm, and the average preoperative weight was 47.27 ± 6.69 kg. The average BMI was 18.42 ± 2.02 kg/m^2^. Among the patients, the number of UIV on T1, T2, T3, T4 and T5 was 4, 9, 19, 16 and 3, respectively. The average fusion segments were 10.90 ± 1.02, and the mean number of instrumented pedicle screws was 15.08 ± 2.03. Table 1 enlisted the demographic and clinical characteristics.

**Table 1:** Demographic characteristics of all subjects

Variables	Cases(*n* = 51)
Gender (female/male)	41/10
Age (y)	14.12 ± 2.05
Risser sign (0/1/2/3/4/5)	9/4/8/7/16/7
Height (cm)	159.33 ± 6.85
Weight (kg)	47.27 ± 6.69
BMI (kg/m^2^)	18.42 ± 2.02
Lenke type (1&2)	36/15
Lumbar modifier(A/B/C)	28/17/6
UIV (T1/T2/T3/T4/T5)	4/9/19/16/3
Fusion segments (n)	10.90 ± 1.02
Pedicle screw (n)	15.08 ± 2.03

Radiographic parameters were shown in Table 2. The average main thoracic Cobb angle was 44.1 ± 7.7°, the mean thoracolumbar/lumbar Cobb angle was 24.1 ± 9.3°, and the mean coronal balance (C7PL-CSVL) was 11.2 ± 7.9 mm, preoperatively. According to ANOVA analysis, there was a significant difference in proximal thoracic Cobb angle, main thoracic Cobb angle, thoracolumbar/lumbar Cobb angle, and thoracic AVT when comparing the preoperative X-ray with immediate postoperative erect X-ray or in preoperative X-ray and final follow-up X-ray. No significant difference was found in the parameters when comparing the immediate postoperative erect and final follow-up review X-rays. With arthrodesis, the main thoracic curve’s correction was approximately 30° and remained stable until the final follow-up. The thoracolumbar/lumbar curve was spontaneously compensated with a correction rate of more than 70%. The preoperative mean thoracic AVT was 33.0 ± 9.1 mm and was significantly improved at immediate erect postoperatively (*P* < 0.001) and at final follow-up (*P* < 0.001). Additionally, no significant difference was observed in either lumbar AVT or coronal balance. Also, no coronal complications were found in the included patients at final follow-up.

**Table 2:** Radiographic parameters of recruited patients preoperatively, at immediate erect and at final follow-up

	Preoperatively	Immediate erect	Final follow-up	*P* value (Pre vs. Im)	*P* value (Pre vs. Final)	*P* value (Im vs. Final)
Proximal thoracic curve	27.2 ± 13.3	16.5 ± 8.2	14.8 ± 7.7	**< 0.001**	**< 0.001**	0.393
Main thoracic curve	44.1 ± 7.7	13.6 ± 7.8	13.8 ± 8.0	**< 0.001**	**< 0.001**	0.886
Lumbar curve	24.1 ± 9.3	8. 2 ± 9.2	7.0 ± 9.2	**< 0.001**	**< 0.001**	0.486
Thoracic AVT	33.0 ± 9.1	12.7 ± 6.8	14.0 ± 7.3	**< 0.001**	**< 0.001**	0.398
Lumbar AVT	13.9 ± 7.1	11.2 ± 7.6	11.4 ± 7.9	0.074	0.102	0.876
CB (C7PL-CSVL)	11.2 ± 7.9	10.7 ± 8.0	9.3 ± 7.7	0.652	0.951	0.697
L1/2 disc wedge angle	2.86 ± 4.32	0.16 ± 3.89	0.98 ± 4.80	**0.002**	**< 0.001**	0.189
L2/3 disc wedge angle	3.53 ± 4.22	0.72 ± 3.47	0.45 ± 3.16	**< 0.001**	**< 0.001**	0.706
L3/4 disc wedge angle	2.71 ± 4.55	0.49 ± 2.46	0.41 ± 3.16	**0.002**	**0.001**	0.904
L4/5 disc wedge angle	0.36 ± 3.40	0.57 ± 3.25	0.48 ± 2.78	0.735	0.843	0.888
L5/S1 disc wedge angle	2.03 ± 2.57	1.44 ± 3.12	0.67 ± 2.29	0.269	**0.011**	0.147
Upper coronal lumbar curve	15.87 ± 6.64	10.33 ± 5.71	5.34 ± 6.16	**< 0.001**	**< 0.001**	**0.010**
Lower coronal lumbar curve	5.08 ± 3.93	5.15 ± 3.70	4.46 ± 3.79	0.917	0.276	0.433

The preoperative disc wedge angles of L1/2, L2/3, L3/4, L4/5 and L5/S1 were 2.86 ± 4.32°, 3.53 ± 4.22°, 2.71 ± 4.55°, 0.36 ± 3.40° and 2.03 ± 2.57°, respectively. At the final follow-up, the disc wedge angle was approximately zero with spontaneous correction of the lumbar curve. However, as for L4/5 disc and L5/S1 disc level, a significant difference between preoperative and postoperative immediate X-rays was detected in each distal unfused disc wedge angle. A significant difference was found at final follow-up in L1/2 disc, L2/3 disc, L3/4 disc, and L5/S1 disc level compared to preoperative X-ray. When considered upper and lower coronal lumbar curve as integral, the upper integral showed significance whether in the postoperative period or at final follow-up. The preoperative upper and lower coronal lumbar curve were 15.87 ± 6.64° and 5.08 ± 3.93°, which were changed to 10.33 ± 5.71° and 5.15 ± 3.70° immediately after surgery. At the final follow-up, the results turned to 5.34 ± 6.16°and 4.46 ± 3.79°, respectively.

**Fig. 2:**
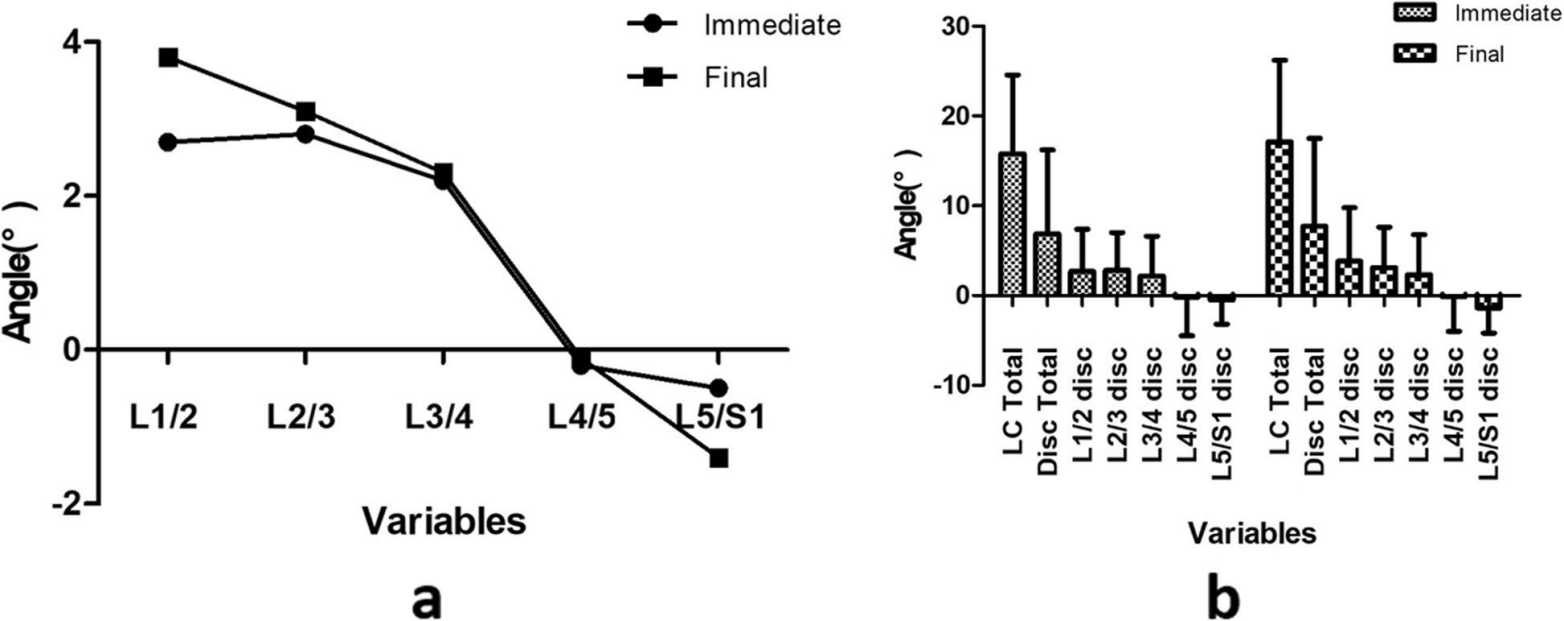
**a** The linear chart demonstrated the compensation ability of each lumbar segment; **b** The distribution of each distal unfused lumbar segment compensation, total lumbar curve correction and total disc compensation

The disc wedge angle variation was calculated to investigate further how each disc level changed and whether this variation was consistent. As shown in Table 3, immediate disc wedge angle variation was 2.70 ± 4.68°, 2.82 ± 4.19°, 2.21 ± 4.38°, − 0.21 ± 4.31° and − 0.59 ± 2.71°, respectively. The disc wedge angle variation at final follow-up was 3.84 ± 5.96°, 3.09 ± 4.54°, 2.30 ± 4.53°, − 0.12 ± 3.89° and − 1.36 ± 2.80°, respectively. Significant differences were found in disc wedge angle variation among each segment after treatment, whether at immediate erect postoperatively or final follow-up. Pairwise comparison showed further significance between L3/4 and L4/5 level postoperatively. As shown in Fig. 2, each disc wedge angle variation presented a decreasing tendency: the further lower the spine, there is the less likely difference. The L1/2 disc and L2/3 disc contributed the most to the lumbar compensation. Besides, by calculating the integral compensation of upper and lower parts, we found the upper coronal lumbar curve play a more significant role of distal unfused lumbar compensation whether in the postoperative period (7.73 ± 8.85° vs − 0.80 ± 5.13°, *P* < 0.001) or at final follow-up (9.22 ± 10.39° vs − 1.49 ± 5.14°, *P* < 0.001), as shown in Table 4. Furthermore, the total disc compensation was added by distal un-instrumented disc wedge angle variation. This compensation accounts for 43.6% at immediate erect and 45.0% at final follow-up.

**Table 3:** Comparison of segmental and regional compensation at immediate erect and final follow-up

	compensation					*P* value				
	L1/2	L2/3	L3/4	L4/5	L5/S1	Overall *P* value	P1	P2	P3	P4
Disc parameters										
Immediate erect minus preoperative	2.70 ± 4.68	2.82 ± 4.19	2.21 ± 4.38	−0.21 ± 4.31	−0.59 ± 2.71	**< 0.001**	0.889	0.461	**0.003**	0.643
Final follow-up minus preoperative	3.84 ± 5.96	3.09 ± 4.54	2.30 ± 4.53	−0.12 ± 3.89	−1.36 ± 2.80	**< 0.001**	0.396	0.372	**0.007**	0.162
*P* value	0.286	0.753	0.718	0.924	0.159	–				
Lumbar curve	compensation					paired t test				
	Upper coronal lumbar curve			Lower coronal lumbar curve		*P* value				
Immediate erect minus preoperative	7.73 ± 8.85			−0.80 ± 5.13		**< 0.001**				
Final follow-up minus preoperative	9.22 ± 10.39			−1.49 ± 5.14		**< 0.001**				

P1: L1/2 vs. L2/3; P2: L2/3 vs. L3/4; P3: L3/4 vs. L4/5; P4: L4/5 vs. L5/S1

**Table 4:** Correlation between each disc wedge angle and lumbar Cobb angle preoperatively and at final follow-up

Preoperative disc wedge angle	Preoperative Lumbar Cobb angle		Disc wedge angle at final follow-up	Lumbar Cobb angle at final follow-up	
	Correlation	*P* value		Correlation	*P* value
L1/2	0.557	**< 0.001**	L1/2	0.518	**< 0.001**
L2/3	0.450	**< 0.001**	L2/3	0.468	**0.001**
L3/4	0.205	0.148	L3/4	0.201	0.158
L4/5	0.364	**0.009**	L4/5	0.067	0.640
L5/S1	−0.289	**0.040**	L5/S1	−0.149	0.298

**Fig. 3:**
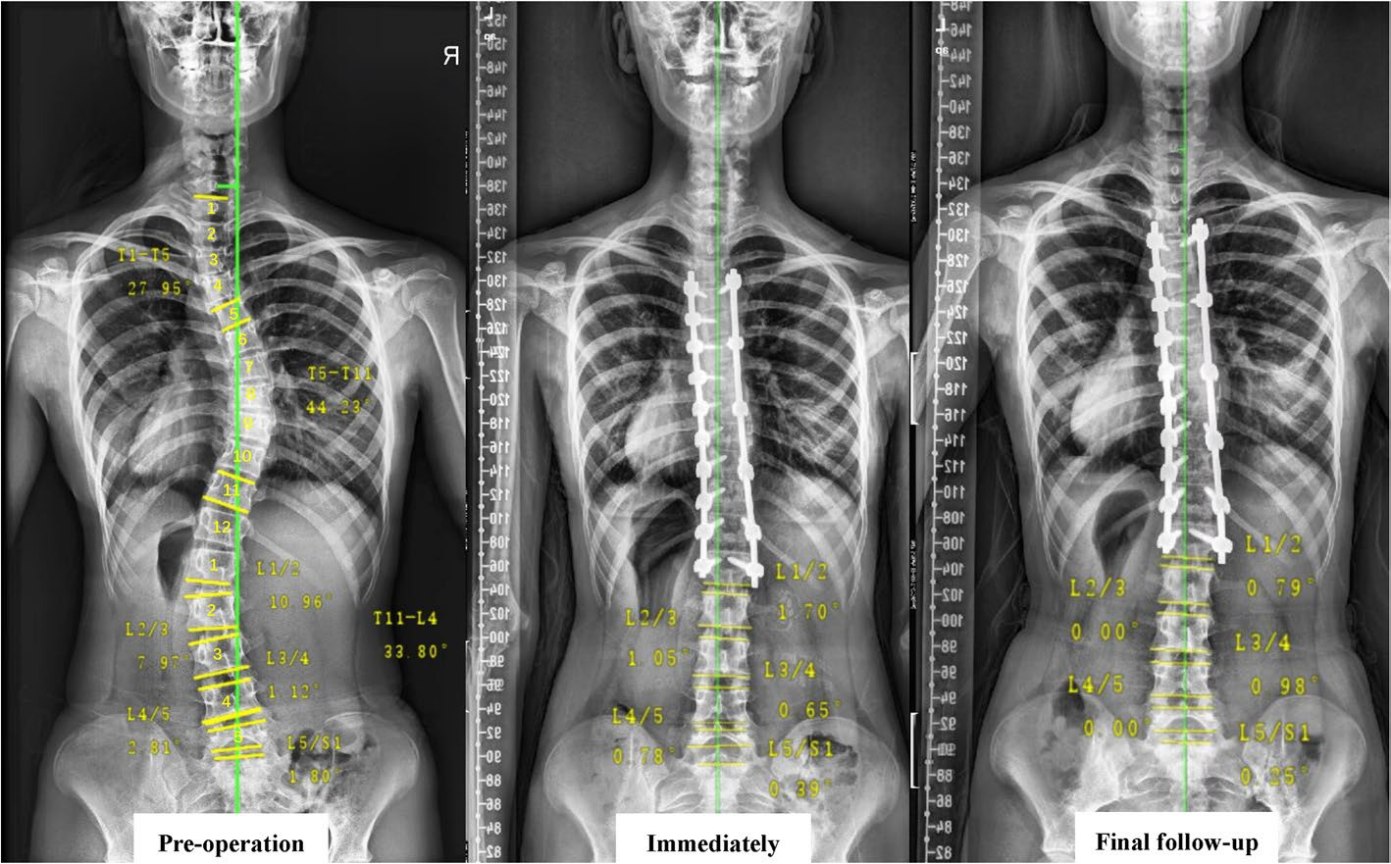
A 15-year-old Lenke 1C AIS patient, female, the LSTV was L1 vertebrae, and the LIV was L1 vertebrae. According to the image, we can see that L1/2 and L2/3 compensate most, while there was nearly no compensation in other segments

As shown in Table 4, a strong association was found between the following disc wedge angle and the TL/L Cobb angle at final follow-up using Pearson correlation statistics: L1/2 wedge angle (*r* = 0.518, *p* < 0.001) and L2/3 wedge angle (*r* = 0.468, *p* = 0.001). Moreover, the correlation of disc compensation and spontaneous lumbar correction at final follow-up showed a similar tendency (Table 5): L1/2 disc compensation (*r* = 0.542, *p* < 0.001) and L2/3 disc compensation (*r* = 0.437, *p* = 0.001). As for the preoperative TL/L Cobb angle, the correlation was not significant in the preoperative L3/4 wedge angle (*r* = 0.205, *p* = 0.148). The typical example was shown in Fig. 3.

**Table 5:** Correlation between each lumbar segment compensation and total lumbar compensation at final follow-up

Disc compensation	Total lumbar compensation	
	Correlation	*P* value
L1/2	0.542	**< 0.001**
L2/3	0.437	**0.001**
L3/4	0.087	0.544
L4/5	0.080	0.579
L5/S1	−0.266	0.060

## Discussion

Previously many studies have been conducted on the compensation of lumbar curvature after thoracic fusion. Koller et al. [14] proposed an accurate prediction model for postoperative SLCC based on the analysis of many prospective STF cases. Danilo et al. [1] conducted a retrospective cohort study of 42 Lenke 1 AIS patients and concluded that the main thoracic curve’s overcorrection might result in less satisfactory results. Matthew et al. [23] indicated that the preoperative push-prone is the best preoperative flexibility radiograph to predict the final lumbar curve measurement. Pasha et al. [7] then developed a decision tree to define criteria for optimal lumbar curve correction following STF in Lenke 1 AIS. Schulz et al. [24] pointed out that optimal postoperative outcomes for STF should include a lumbar Cobb angle less than 26 °, coronal balance 2 cm or less, deformity-flexibility quotient less than 4, lumbar correction more than 37%, and trunk shift less than 1.5 cm. However, these studies only focus on overall compensation behavior and aim to improve clinical strategies.

In this study, we calculated each disc wedge variation of distal unfused lumbar segments to further elucidate the characteristics of spontaneous compensation of the lumbar curve after STF. The results showed that the proximal two segments at level L1/2 and L2/3 accounted for most total compensation. The distal unfused lumbar segments provided the more distal the segment, the less compensation. Furthermore, we found that total disc compensation consisted of less than half of the total postoperative lumbar curve compensation. This phenomenon may indicate that the lumbar curvature is often affected and includes the thoracic vertebrae, such as T10, T11 and T12. However, since all of our cases chose L1 as LIV, our study did not further investigate the fused thoracic discs, we focused attention on the unfused lumbar segments. As shown in Fig. 2, the compensation ability of the lumbar segments showed a decreasing tendency, with a major role being played by the proximal adjacent lumbar curve. Moreover, our integral analysis indicated that the upper coronal lumbar curve was responsible for most of the compensation, which was consistent with the opinion of Na et al. [25]. They were the first to divide the lumbar curve into the proximal and distal curves by their respective lumbar apex and concluded that looking at the proximal lumbar curve flexibility might be an alternative indicator for measuring the lumbar flexibility in MT-AIS patients treated by STF. We believe that the characteristics of residual lumbar curve after STF may be closely associated with the adding-on phenomenon and may provide evidence when choosing the correct LIV.

Then, what is the reason for the non-uniformity of unfused distal segment compensation? We believed that the flexibility of the distal unfused segments might be different. Zhao et al. [26] analyzed the characteristics of cobb angle distribution in the Lenke 5C AIS patients. They found that the disc angles had symmetric distribution in the main thoracolumbar/lumbar curve, while the distal segment is more flexible. The thoracolumbar/lumbar curve’s apex was often L1 or L2 vertebrae, and its distal segments may correspond to the L1/2 and L2/3 segments that were consistent with our study results. Na et al. [25] also found that the lumbar apex of 28 main thoracic curve patients was between L2 and L3, and concluded that the curve flexibility of the proximal lumbar area could be meaningful. Jansen et al. [27] also concluded that in STF patients, the most correction was made in the upper part of the lumbar curve, while the distal lumbar curve seemed to be more rigid and less important in spontaneous curve correction. In addition, another reasonable hypothesis may be the mechanical effect of posterior fusion with the pedicle screw. This phenomenon was similar to complications on the sagittal plane, such as PJK [28, 29] and DJK [30], which we believed could result from stress concentration on the adjacent segments. Meric et al. [12] have shown that facet joint degeneration is significant at the upper two levels adjacent to the LIV when performing STF. Furthermore, this may be explained by the principle of load-sharing, that when arthrodesis was applied, the posterior fixation conducted most of the forces to the lowest instrumented vertebrae. Furthermore, when this force is overloaded, the stress could be conducted to the most adjacent segments. This has related to coronal complications, which could lead to complications, including adding-on phenomenon and coronal imbalance.

Therefore, when planning surgical treatment strategies, the characteristics of unfused lumbar segments should be carefully considered. Inappropriate curve selection and excessive thoracic correction have been identified as the most common etiologies of coronal imbalance [4, 31, 32]. Meanwhile, numerous studies [8, 21, 31–33] have demonstrated improper placement of the LIV is also an independent risk factor. It is also important to realize the heterogeneity of spontaneous compensation of unfused lumbar segments. When fusing the thoracic curvature, the overall compensation ability of the lumbar curvature and the heterogeneity of compensation to avoid excessive compensation at the proximal end should be considered.

Even though our study focused on the residual lumbar curve segmental characteristics in Lenke 1 and 2 AIS patients who were performed STF, several limitations should be considered. First, we only included patients whose LIV was L1 vertebrae for the homogeneity analysis of disc compensation. Further researches on other LIV selection and comparison should be performed. Second, only coronal position data were studied in our research but not a sagittal plane, and there was no specific analysis of related complications. Finally, this was a single-center study, and multi-centric research should be conducted to further validate the results.

## Conclusion

The residual lumbar curve can be corrected spontaneously with the thoracic curve correction after posterior thoracic fusion in Lenke 1 and 2 AIS patients. When selecting L1 as the lowest instrumented vertebrae, the compensation of distal unfused lumbar segments showed a declining tendency to contribute to the compensation; with the immediately adjacent L1/2 and L2/3 disc contributed most in this compensation.

## Data Availability

The data that support the findings of this study are available from Changhai Hospital, China, but restrictions apply to the availability of these data, which were used under license for the current study, and so are not publicly available. Data are however available from the authors upon reasonable request and with permission of Changhai Hospital, China.

## Abbreviations

AIS: Adolescent idiopathic scoliosis
STF: Selective thoracic fusion
SLCC: Spontaneous lumbar curve correction
LIV: Lowest instrumented vertebrae
UIV: Upper instrumented vertebrae
TAVT: Translation of thoracic apex
C7PL: The cervical 7 vertebrae plumb line
LAVT: Translation of thoracolumbar/lumbar apex
CSVL: The center sacral vertical line
SD: Standard deviation
